# Formation of Cluster‐Structured Metallic Filaments in Organic Memristors for Wearable Neuromorphic Systems with Bio‐Mimetic Synaptic Weight Distributions

**DOI:** 10.1002/advs.202307494

**Published:** 2023-12-12

**Authors:** Uihoon Jung, Miseong Kim, Jaewon Jang, Jin‐Hyuk Bae, In Man Kang, Sin‐Hyung Lee

**Affiliations:** ^1^ School of Electronics Engineering and School of Electronic and Electrical Engineering Kyungpook National University 80 Daehak‐ro, Buk‐gu Daegu 702‐701 Republic of Korea

**Keywords:** artificial synapse, metallic filament, organic memristor, synaptic weight, wearable neural network

## Abstract

With increasing demand for wearable electronics capable of computing huge data, flexible neuromorphic systems mimicking brain functions have been receiving much attention. Despite considerable efforts in developing practical neural networks utilizing several types of flexible artificial synapses, it is still challenging to develop wearable systems for complex computations due to the difficulties in emulating continuous memory states in a synaptic component. In this study, polymer conductivity is analyzed as a crucial factor in determining the growth dynamics of metallic filaments in organic memristors. Moreover, flexible memristors with bio‐mimetic synaptic functions such as linearly tunable weights are demonstrated by engineering the polymer conductivity. In the organic memristor, the cluster‐structured filaments are grown within the polymer medium in response to electric stimuli, resulting in gradual resistive switching and stable synaptic plasticity. Additionally, the device exhibits the continuous and numerous non‐volatile memory states due to its low leakage current. Furthermore, complex hardware neural networks including ternary logic operators and a noisy image recognitions system are successfully implemented utilizing the developed memristor arrays. This promising concept of creating flexible neural networks with bio‐mimetic weight distributions will contribute to the development of a new computing architecture for energy‐efficient wearable smart electronics.

## Graphical abstract

Flexible neuromorphic systems for complex computations are implemented using organic memristors with bio‐mimetic synaptic weights. In the device, the cluster‐structured filaments are achieved by optimizing the polymer conductivity, which leads to the continuous memory states. The systems showed stable computing performances including ternary logic operators and noisy image recognition systems.

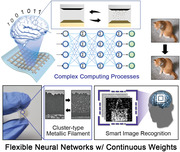

## 1 Introduction

With the advancement of information technologies, such as the Internet of Things and artificial intelligence, there is an increasing demand for smart wearable systems capable of rapidly processing massive amounts of data with low energy consumption.^[^ ^1^, ^2^, ^3^ ^]^ A traditional computing architecture based on the von Neumann system has been presented to be unsuitable for smart wearable electronics due to its bottlenecks problems and poor energy efficiency.^[^ ^4^, ^5^ ^]^ Recently, neuromorphic hardware systems mimicking the behaviors of the human brain have received much attention as a new computing architecture capable of overcoming the von Neumann bottlenecks. In such computing systems, the biological synaptic connections between neurons are replicated using memory components for parallel computation, and the system performance is primarily determined by the characteristics of the memory cells.^[^ ^5^, ^6^, ^7^ ^]^ To date, various types of the flexible memory devices, including the organic transistors^[^ ^8^, ^9^ ^]^ and memristors,^[^ ^10^, ^11^, ^12^, ^13^ ^]^ have been explored for the construction of wearable neural networks. However, previously developed flexible neuromorphic systems have only been utilized to recognize basic patterns. This limitation primarily stems from the constrained number of non‐volatile memory states of the synaptic memory. To create hardware neural networks that can effectively classify intricate images with a level of accuracy comparable to biological systems, it becomes imperative to attain analog memory states featuring non‐volatile characteristics in the memory cell.^[^ ^8^ ^]^

Solution‐processed organic memristors have been demonstrated as a promising memory component for flexible neural networks, considering their mechanical flexibility and scalability.^[^ ^11^, ^12^, ^14^, ^15^, ^16^ ^]^ Particularly, the organic memristors based on the filamentary conduction have been evaluated as promising candidates for synaptic components in neuromorphic systems, owing to the high current ratio between the high and low resistance states (HRS and LRS) and the stable retention performance for non‐volatile memory characteristics.^[^ ^11^, ^12^, ^15^ ^]^ In the filamentary based organic memristors, the metallic filaments are formed by the electric stimuli, resulting in the resistive switching features of the devices. Additionally, multilevel conductance states can be achieved in the devices by precisely controlling the filament thickness.^[^ ^17^, ^18^ ^]^ However, in such devices, the resistive switching phenomenon achieved by the abrupt growth of metallic filaments is difficult to control, making it challenging to linearly regulate the conductance level, similar to a biological counterpart. These challenges in tuning the conductance state inherently restrict the memory states of the device, consequently deteriorating the accuracy of the constructed neuromorphic systems. Several studies have been conducted thus far to investigate strategies for linearly controlling the conductance of memristors.^[^ ^11^, ^19^, ^20^ ^]^ In order to achieve gradual growth of metallic filaments, progressively varying electric pulses were employed for resistive switching within the devices. While the device conductance was linearly controlled at the LRS, intricate external systems were necessary to precisely engineer the pulses. Additionally, the number of non‐volatile memory states was limited due to the unstable characteristics of the immature filaments. In recent times, a gradually alteration in conductance was reported in the memristors that incorporate a conducting layer instead of an insulating film.^[^ ^21^, ^22^, ^23^, ^24^ ^]^ However, the on/off conductance ratio and the resultant memory levels in the devices involving a conducting film were significantly restricted due to the substantial leakage current. Moreover, the dynamics of metallic filament growth in a conducting medium, which give rise to gradual resistive switching in such devices, have yet to be elucidated. Clarifying a physical image for filament growth in the conducting medium and creating an organic memristor with the linearly tunable conductance are both crucial steps toward the goal of developing wearable neuromorphic systems analogous to the human brain.

**Figure 1:**
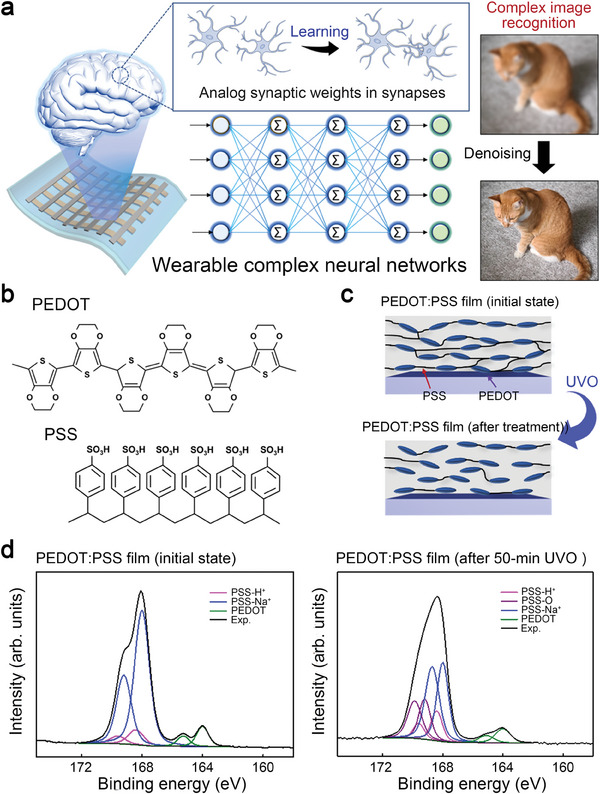
Concepts and polymer material properties for wearable complex neural networks consisting of organic memristors with bio‐mimetic synaptic weight distributions. a) A schematic presenting the complex neural networks with bio‐realistic synaptic weight distributions. b) A chemical structure of the conducting polymer, Poly (styrenesulfonate)‐doped poly (3,4‐ethylenedioxythiophene) (PEDOT:PSS). c) Schematics of the ultraviolet‐ozone (UVO) treatment effects on the PEDOT:PSS film structure. d) XPS spectra of the PEDOT:PSS films with and without UVO treatment. The treatment duration lasted for 50 min.

In this study, we demonstrated the flexible organic memristor with the facilely tunable conductance, similar to a biological synapse, for the realization of wearable neuromorphic systems capable of facilitating intricate computing processes, as shown in **Figure** **1**a. To clarify a physical picture of the gradual resistive switching in organic memristors, we investigated the conductivity‐dependent dynamics of metallic filament growth within a polymer medium. With an increase in the conductivity of the polymer medium, the filament structure underwent a transition from a bridge type to a cluster type, resulting in an enhanced linearity for resistive switching in the device. Based on this physical understanding, we have developed a flexible organic memristor governed by cluster‐structured filaments, which consist of a polymer medium with low leakage current. The developed flexible device showed the superior linearity in potentiating the conductance via the simple electric stimuli, and stable synaptic functions that closely resemble the biological counterparts. A large number of conductance states with non‐volatile characteristics were effectively presented in the developed synaptic memory component. Furthermore, the flexible neural networks for computing ternary logics were demonstrated using the developed memristor arrays, and our memristor exhibited strong potentials for constructing smart neuromorphic systems that can recognize complex images irrespective of noise.

## 2 Results and Discussion

Poly (styrenesulfonate)‐doped poly (3,4‐ethylenedioxythiophene) (PEDOT:PSS) is a promising conducting polymer as an active medium for the organic memristor based on the metallic filament^[^ ^22^, ^23^ ^]^ (see Figure 1b). For engineering the conductivity of the PEDOT:PSS film, we performed the ultraviolet‐ozone (UVO) treatment for the film.^[^ ^24^ ^]^ The UVO treatment is widely used to fabricate the commercial organic electronics including the organic‐light emitting diodes and solar cells.^[^ ^25^, ^26^ ^]^ As shown in Figure 1c, chemical bonds in the PEDOT:PSS film can be broken down by the UVO treatment, leading to a reduction in the film's conductivity. The PSS is decomposed and oxidized through the UVO treatment; thus, it cannot act as a functional dopant to support charge flow, leading to a reduction in film conductivity. Figure 1d presents the XPS spectra for validating the impact of the UVO treatment on the chemical bonds within a PEDOT:PSS film. The spectra were all fitted using a Gaussian and Lorentzian convolution. After the UVO treatment for 50 min, a binding energy peak at ≈169 eV, indicative of sulfur atoms binding with oxygen, became evident in the film, meaning the decomposition of the PSS‐Na^+^ and PSS‐H^+^ bonds.^[^ ^24^, ^27^ ^]^ Since the decomposed PSS was not serving as a functional dopant to facilitate a charge flow, the UVO treatment effectively tuned the conductivity of the PEDOT:PSS film. Note that the presence of Na^+^ residues are attributed to the use of the Na_2_S_2_O_8_ oxidizing agent during the polymerization of PEDOT:PSS.^[^ ^27^ ^]^

**Figure 2:**
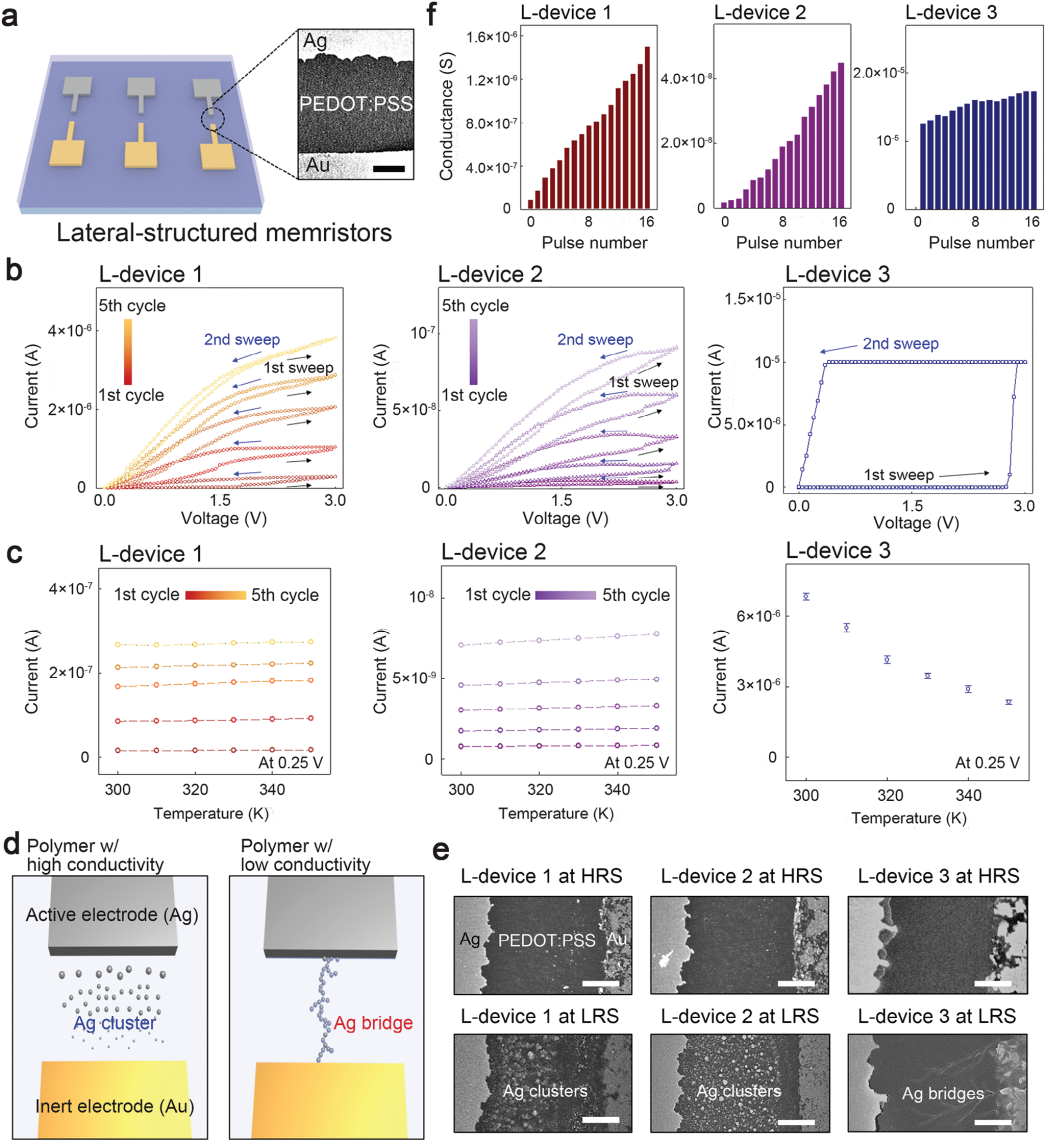
Effects of polymer conductivity on filament growth and resulting resistive switching properties in organic memristor. a) A schematic of the lateral‐structured organic memristor for exploring the effect of polymer conductivity. An inset image depicts the device configuration observed by the field‐emission scanning electron microscope (FE‐SEM) (scale bar, 150 nm). b) The current–voltage curves of the lateral‐structured memristors under the various ultraviolet‐ozone treatment conditions: L‐device 1 with no treatment, L‐device 2 with 50‐min treatment, and L‐device 3 with 100‐min treatment. All measurements were performed using a compliance current of 10^−5^ A. c) Conductance variations at the low resistance states (LRS) of the devices at temperature ranges from 300 to 350 K. d) A schematic illustrating the filament structure of the organic memristor based on the polymer conductivity. e) FE‐SEM images of the active regions of the devices at the high resistance state (HRS) and LRS (scale bar, 150 nm). f) Conductance variations in the devices when subjected to successive 2.8 V‐voltage pulses with 100‐µs width.

To study the effect of polymer conductivity on the growth of metallic filaments, we prepared three lateral‐structured memristors. These devices were composed of PEDOT:PSS films that were subjected to various UVO treatment conditions: L‐device 1 with no treatment, L‐device 2 with 50‐min UVO treatment, and L‐device 3 with 100‐min UVO treatment (see **Figure** **2**a). Figure 2b shows the current–voltage (*I*−*V*) characteristics of the devices. All devices were measured at a compliance current of 10^−5^A during the writing process, which consisted of positive voltage sweeps. The initial current level of the device was effectively reduced according to the UVO treatment duration, which is consistent with the results demonstrated in Figure 1d. In a reference device without UVO treatment (L‐device 1), the resistive switching behaviors were achieved progressively, in line with the findings from previous studies.^[^ ^22^, ^23^ ^]^ Additionally, the device (L‐device 2) with a relatively high resistance in the initial state also exhibited the gradual resistance switching characteristics, similar to the reference device. The resistance values of both L‐device 1 and L‐device 2 increased linearly with the sweep number. However, in the device with the lowest leakage current (L‐device 3), abrupt resistive switching was observed, akin to a typical organic memristor containing an insulating medium.^[^ ^28^, ^29^ ^]^ During the erasing processes involving negative voltage sweeps (see Figure S1, Supporting Information), the resistive switching tendency of each device was consistent with that observed during the writing process. This suggests that the growth of metallic filaments, which dictates the resistive switching characteristics of the devices, is significantly influenced by the conductivity of the polymer medium.

Following the writing processes, we examined the conduction mechanism to estimate the filament structure of each device. All the devices exhibited a positive linear relationship between current and voltage values (see Figure S2, Supporting Information), indicating either Ohmic conduction via a bridge‐structured metallic filament or direct tunneling conduction via cluster‐structured filaments.^[^ ^30^ ^]^ Figure 2c depicts the relationship between temperature and resistance values subsequent to the writing processes conducted on each device. The resistances of each device were investigated at a reading voltage of 0.25 V while the temperature was increased from 300 to 350 K. For L‐device 1 and L‐device 2, the LRS conductance was found to be almost temperature independent, indicating direct tunneling.^[^ ^30^, ^31^ ^]^ In contrast, the LRS conductance of L‐device 3 was reduced as the temperature increased, which is accordance with the properties of the organic memristors following the Ohmic conduction over a metallic bridge.^[^ ^32^ ^]^ These results indicate a strong dependence of the metallic filament growth on the polymer conductivity of the organic memristor. Figure 2d illustrates the switching mechanisms of the device according to the polymer conductivity. When the polymer conductivity for charge drift is sufficiently high, metallization of the metal cations occurs within the polymer medium, leading to the formation of cluster‐structured filaments in the device. On the contrary, when the conductivity of the polymer medium was reduced to the level of an insulator, the filament growth commenced from the interface between the electrode and the polymer medium due to the low charge mobility, resulting in the formation of a bridge structured filament.^[^ ^33^, ^34^ ^]^

To directly verify the metallic filament structures in the devices with varying polymer conductivity, the active area of each device was examined in both the HRS and the LRS using field emission electron scanning microscopy, as depicted in Figure 2e. In L‐device 1 and L‐device 2, the presence of cluster‐structured filaments was clearly observed in the LRS, whereas L‐device 3 exhibited dendrite‐like filaments bridging the electrodes. This indicates that the dynamics of the filament growth in the organic memristors can be effectively controlled by the medium's conductivity, and it aligns with the outcomes derived from the electrical characteristics of the devices (see Figure 2c,d).

Subsequently, we conducted a more comprehensive analysis of the conductance linearity in the writing and erasing processes of the devices following the different kinetics for the metallic filament growth, as shown in Figure 2f and Figure S3 (Supporting Information). For the writing and erasing processes for the devices, 2.8 V‐ and −2.2 V‐pulses of 100‐µs width were used respectively. The organic memristors with the cluster‐type filaments (L‐device 1 and L‐device 2), exhibited a continuous increase (or decrease) during positive (or negative) voltage sweeps, similar to the successive weight tuning in a biological synapse. However, in the case of the device relying on bridge‐type filaments (L‐device 3), the conductance underwent relatively nonlinear changes, rendering it unsuitable for practical neuromorphic systems.^[^ ^8^ ^]^ This means that by regulating the polymer conductivity related to the metallic filament structure, it is possible to attain both the simply tunable conductance and low leakage current for the high current ratio, which are well‐suited for achieving the numerous memory states, in the organic memristor.

**Figure 3:**
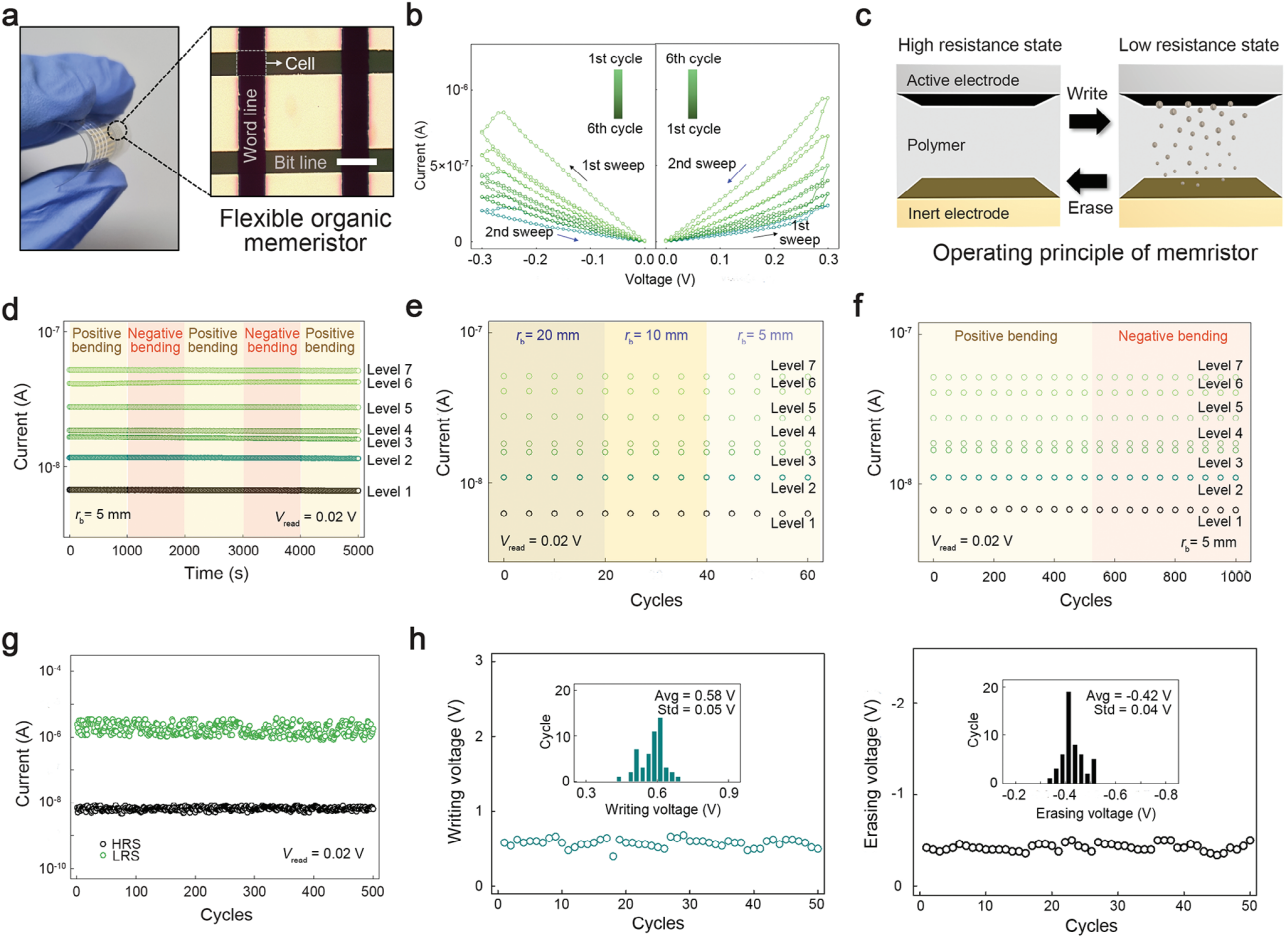
The electrical and mechanical characteristics of the developed vertically‐structured flexible memristor. a) A photograph depicting the vertically‐structured organic memristors prepared on a flexible substrate. An inset image presents the array structure observed by the microscope (scale bar, 70 µm). b) The current–voltage characteristics of the device. c) A schematic exhibiting the operating principle for resistive switching in the device. d) The memory retention characteristics of the device. The device was subjected to repeated compressive and tensile stresses with a bending radius (*r*_b_) of 5 mm to confirm its mechanical stability. e) Resistive switching characteristics of the device at positive bending states for different *r*_b_ values. f) Mechanical and g) electrical cycle tests for evaluating the endurance performance of the device. h) Dispersions in switching (writing and erasing) voltages in the device for 50 successive cycles.

From a physical understanding of the cluster‐type filament growth in organic memristors, we developed a vertically structured flexible memristor, suitable for emulating the analog synaptic weights, as depicted in **Figure** **3**a. To achieve the gradual growth of metallic filaments in organic memristors consisting of the insulating polymer with the high current ratio, we applied the UVO treatment for the optimized duration (50 min) to a polymer (PEDOT:PSS) film of the device (see Figure S4, Supporting Information). With an increase in the UVO treatment time, the leakage current decreased, and resistive switching was more abruptly achieved. When the UVO treatment was performed on the polymer film for 50 minutes, the device exhibited progressively resistive switching behaviors with the high current ratio under low leakage current conditions. Note that, the polymer film with the 120‐min UVO treatment showed the high leakage current level, due to the degradation in the film structure.^[^ ^27^ ^]^ Additionally, to effectively reduce the device's leakage current, we patterned the conducting polymer film before the UVO treatment. In patterning the polymer film, we used the self‐organization method (see Figure S5, Supporting Information). The organic memristor consisting of the non‐patterned polymer film showed a high leakage current, irrespective of the UVO treatment (see Figure S6, Supporting Information). Figure 3b shows the *I*–*V* curves of the device measured during the consecutive voltage sweeps. The device exhibited gradual and continuous resistive switching characteristics, which were achieved in a reversible bipolar mode. After the positive voltage sweeps for the writing process, the current flow for the device was governed by the direct tunneling mechanism attributed to the cluster‐type filaments (see Figure S7, Supporting Information). In addition, the conductance level of the device at the HRS (≈3.3 × 10^−7^ S) was highly lower than that (≈6.7 × 10^−4^ S) of the conducting polymer‐based memristors without the UVO treatment (see Figure S4, Supporting Information). This means that the organic memristor relying on the cluster‐type filaments was successfully developed utilizing the optimized insulating polymer medium with a low leakage current, as illustrated in Figure 3c. Note that the device displayed abrupt resistive switching when the writing voltage was high enough to form complete filaments connecting the electrodes (see Figure S8, Supporting Information).

For constructing the hardware neural networks, close to a biological system, it is essential to achieve the non‐volatile memory properties that endure for several hours,^[^ ^8^, ^35^, ^36^ ^]^ in the synaptic device. Figure 3d illustrates the memory retention performance of the device under varying conductance levels during the writing processes. Each conductance state was measured under the repeated positive and negative bending stresses with a radius of 5 mm, to confirm the mechanical flexibility of the device. All the memory states of the memristor were maintained without any deterioration, during the bending state. Moreover, our device was stably operated in the harsh condition (with a relative humidity of 80%, at 50°C), as shown in Figure S9 (Supporting Information). This indicates that the device can serve as a stable synaptic cell in the wearable neuromorphic systems. To further evaluate the mechanical durability of the device, we carried out the several bending tests for that, as shown in Figure 3e,f. Regardless of the bending stresses associated with varying radius values, the device exhibited robust non‐volatile memory characteristics. The memory states of the device also remained stable under repeated compressive and tensile stresses at a radius of 5 mm, indicating the superior mechanical flexibility.^[^ ^37^ ^]^ Figure 3g shows the cycle test results for the device measured at the successive voltage sweeps. For the writing and erasing processes in the measurement, we applied relatively high voltage sweeps ranging from 0 to 0.6 V and from 0 to −0.5 V, respectively, to impose sufficient stress for testing the electrical endurance of the device. Our device demonstrated 500 cycles of stable resistive switching performance, comparable to that of other practical memory devices.^[^ ^3^, ^38^ ^]^

In order to confirm the reproducibility of the device's resistive switching, we analyzed the temporal variations in writing and erasing voltages during the switching cycles consisting of the identical voltage sweeps as those in Figure 3g (see Figure 3h). The variation values were calculated as the ratio of the standard deviation to the mean value. The temporal variations for writing and erasing were ≈0.09 and −0.10, respectively, suggesting the reproducible resistive switching characteristics, similar to those of the typical memristors.^[^ ^39^, ^40^ ^]^ Furthermore, when analyzing the dispersion of switching voltages among the eight different devices on the single substrate, only slight variations were observed (see Figure S10, Supporting Information). Given that the introduction of localized ion injection can significantly enhance the reproducibility and device‐to‐device uniformity of the organic memristor,^[^ ^41^, ^42^ ^]^ it is reasonable to conclude that the developed flexible memristor is suitably reliable for practical applications.

**Figure 4:**
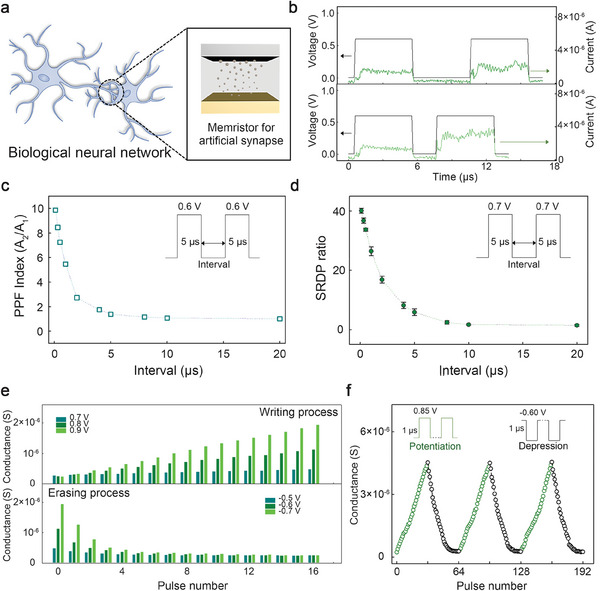
Synaptic characteristics mimicked in the developed organic memristor. a) A schematic of the device acting as an artificial synapse. b) Paired‐pulse facilitation (PPF) confirmed in the device by two successive 0.6‐V pulses with 5‐µs width. c) PPF index and d) spike‐rate‐dependent plasticity of the device, as a function of the time interval between repeated pulses. e) Spike‐number‐dependent plasticity observed in the device based on pulse amplitude (0.7, 0.8, and 0.9 V). The width of each pulse was set to 1 µs, and the intervals between the pulses were fixed as 10 µs. f) The multilevel conductance levels of the device operating in pulse mode.

For realizing the bio‐realistic neuromorphic systems operating in a simple pulse mode, it is crucial to replicate the synaptic functions in the synaptic memory component.^[^ ^43^, ^44^ ^]^ As shown in **Figure** **4**a, we investigated the synaptic functions of the device in response to the electric stimuli. Short‐term plasticity (STP), long‐term plasticity (LTP), and STP‐to‐LTP transition features for biological systems can be selectively replicated by adjusting a pulse amplitude or width as the memory volatility in organic memristors is often governed by the filament stability.^[^ ^45^, ^46^ ^]^ When an electric stimulus of 0.5 V with 5.5‐µs width was applied to our organic memristor, the device exhibited volatile resistive switching behavior due to immature filaments, and the increased conductance was exponentially relaxed back to its initial state (see Figure S11, Supporting Information). This is analogous to STP.^[^ ^7^, ^47^ ^]^ However, at a relatively large stimulus (0.8 V‐pulse with 5.5‐µs width), the device exhibited the non‐volatile memory characteristics, attributed to the stable filaments, similar to LTP (see Figure S12, Supporting Information). As a non‐volatile memory, the writing and erasing times of the device were ≈1.1 µs and 1.8 µs, respectively. Note that the switching times for the organic memristors can be significantly reduced by engineering the amplitude of an electric stimulus.^[^ ^17^, ^18^ ^]^

To further represent the STP characteristics of our memristor, two successive 0.6‐V electric stimuli with 5‐µs width were applied to the device, as shown in Figure 4b. When exposed to electrical stimuli, the device exhibited an elevated current level, an excitatory post‐synaptic current (EPSC). Notably, the EPSC recorded during the second stimulus (A_2_) exceeded that of the first pulse (A_1_), suggestive of the manifestation of a key feature of STP known as paired‐pulse facilitation (PPF).^[^ ^48^ ^]^ Furthermore, it was observed that the EPSC showed greater enhancement when the interval between the stimuli was shorter, similar to a biological synapse. Figure 4c presents the PPF index calculated from the ratio of A_2_ to A_1_ in response to a variable time interval between stimuli. The same pulses as those in Figure 3b were employed. As the time interval decreased from 20 to 0.1 µs, the index value increased from 1.01 to 9.85. This indicates that the device exhibited stable STP characteristics.^[^ ^49^, ^50^ ^]^

Furthermore, we analyzed the spike‐dependent synaptic learning functions including spike‐rate‐dependent plasticity (SRDP) and spike‐number‐dependent plasticity (SNDP) in our flexible memristor. To measure the SRDP characteristics of the device, we used two sequential 0.7‐V pulses, each lasting 5 µs, and changed the time interval between the pulses from 20 to 0.1 µs. We then estimated the SRDP gain as the ratio of the changed conductance to the initial conductance, according to the time interval (see Figure 4d). With decrease in the time interval, the SRDP gain increased from ≈1.80 to ≈40.43, being indicative of a history‐dependent synaptic plasticity that contributes to an energy‐efficient learning process. Note that the gain values obtained from the SRDP measurement were decreased when the lower voltage pulses (0.6 V) were utilized (see Figure S13, Supporting Information). Figure 4e shows the SNDP features of the device investigated in the three different pulse amplitude conditions (0.7, 0.8, and 0.9 V for writing and −0.5, −0.6, and −0.7 V for erasing). For each condition, the successive voltage pulses with the 1‐µs width were used. As the pulse number for the positive voltage increased, the device conductance increased linearly, and the increased conductance value was effectively controlled by the pulse amplitude. Although the device showed the relatively nonlinear decrease in the conductance at the negative voltage conditions, it is possible to effectively enhance the linearity during the erasing process by optimizing the voltage conditions to suppress the thermal fuse effect in the filaments.^[^ ^51^, ^52^ ^]^ In the pulse condition with the high magnitude (1.2 V), the device showed the abrupt resistive switching behaviors according to the pulse number (see Figure S14, Supporting Information), which is accordance with the result in Figure S8 (Supporting Information).

To emulate the weight distributions of a biological synapse, we precisely tuned the conductance of the device by utilizing the simple electric stimuli, as shown in Figure 4f. The device was potentiated and depressed utilizing 0.85‐V and −0.60‐V pulses, respectively, with the width of each pulse being 1 µs. During the potentiation and depression processes, the 32 different conductance states were clearly achieved in the device. Particularly, in the potentiation process, the nonlinearity factor^[^ ^53^ ^]^ for the conductance change was found to be ≈0.1, which is highly superior to the other memristors tuned by the simple pulse condition (see Figure S15, Supporting Information).^[^ ^11^, ^54^, ^55^, ^56^, ^57^, ^58^, ^59^ ^]^ This implies that the developed flexible memristor possessed the bio‐mimetic synaptic conductance, which bodes well for effectively replicating the numerous weight levels of a synapse. Given this easily controllable conductance state, akin to a biological system, it is conceivable that our device could be employed to implement high‐performance complex hardware neural networks.^[^ ^7^, ^8^ ^]^

**Figure 5:**
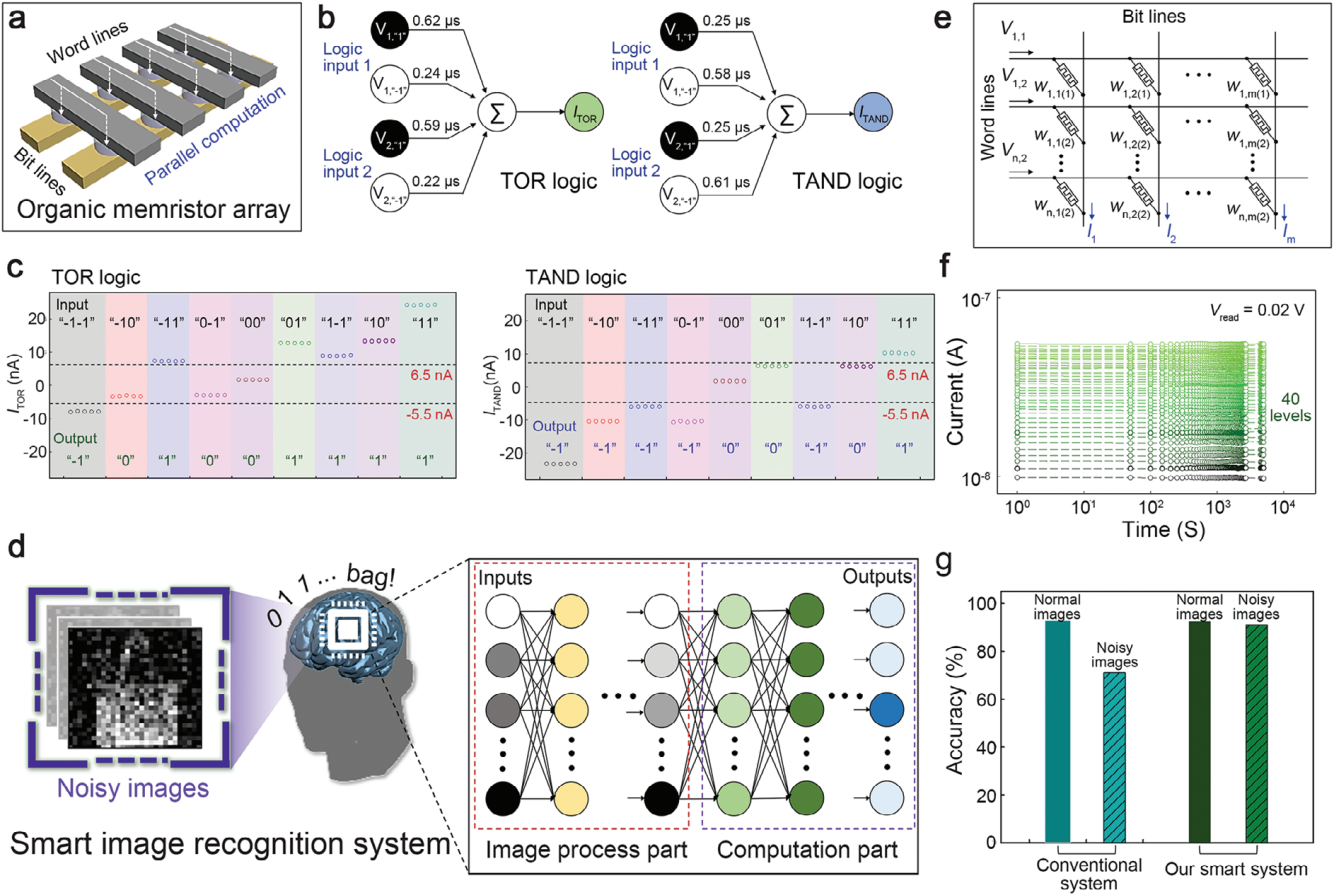
Potentials of the developed organic memristor in practical and complex neuromorphic systems. a) A conceptual illustration of the crossbar arrays of the organic memristors. b) A hardware neural network developed for the ternary logic operation (TOR and TAND). c) The TOR and TAND logic operations demonstrated in the hardware neural networks based on memristor arrays. d) The constructed smart hardware neural network for accurately recognizing noisy fashion images. e) The memristor crossbar arrays for the smart hardware neural network. f) The multilevel memory states achieved by the synapse cell consisting of the two memristors. g) The image recognition accuracy after 40 epochs of training in conventional and smart hardware neural networks consisting of the organic memristor arrays.

To verify the capabilities of the developed flexible memristor as a synaptic memory component, we prepared the memristor arrays for parallel computation, as shown in **Figure** **5**a. We fabricated the hardware neural networks for the ternary logic operators, as shown in Figure 5b. The ternary system is the next‐generation computing architecture capable of transmitting and storing significantly more data than conventional binary systems.^[^ ^60^ ^]^ Despite considerable effort has been made to develop the ternary computing systems, it is still difficult to fabricate the stable ternary logic operators with high energy efficiency. To construct the TOR and TAND logic operators with high energy efficiency, we trained a 4×2 crossbar structured array composed of our organic memristors with the high multilevel capabilities (Figure S16, Supporting Information). Each logic operator consisted of four input neurons and a single output neuron connected via the four memristor acting as a synapse. To handle a logic input, two different input neurons were employed: one for a positive input value (“1”) and another for a negative input value (“−1”). The 100‐ns voltage pulses of 0.02, −0.02, and 0.001 V were applied to the word lines for the input neurons as a logic input of “1”, “−1”, and “0”, respectively, For the logic input “1”, pulses of 0.02 V and 0.001 V were applied to the positive and negative input neurons, respectively. Conversely, for the logic input of “−1”, 0.001 V, and −0.02 V pulses were applied to the positive and negative neurons, respectively. Additionally, for the logic input “0”, both the positive and negative neurons were biased by a voltage pulse of 0.001 V (see Figure S16, Supporting Information). While the two logic inputs were applied to the four neurons, the current values of the bit lines were checked for the TOR and TAND logics. When the current level of the bit line exceeded 6.5 nA (or fell below −5.5 nA), the logic output was set to 1 (or −1); otherwise, it was considered as 0. As shown in Figure 5c, our neural network was operated stably as a ternary logic system for TOR and TAND in all input scenarios (refer to Table S1, Supporting Information), which demonstrates that our flexible memristor possesses the high capabilities required to realize practical smart wearable systems. In the system, the average energy consumption for computing the TOR and TAND logics was measured to be ≈0.0223 fJ and 0.0220 fJ, respectively, which are significantly lower than those of other ternary logic circuits.^[^ ^60^, ^61^ ^]^ Moreover, our system operates at high speeds while dissipating no static energy. Consequently, it can be thought that the developed neural network is more suitable for the energy efficient smart computing applications than other ternary systems.

To further analyze the potentials of our flexible memristor for the practical systems, the numerical simulation was carried out to construct the smart image recognition system utilizing our devices (see Figure 5d). We designed the hardware neural network that can stably classify the noisy images based on a dataset from the Fashion Modified National Institute of Standards and Technology (MNIST).^[^ ^11^, ^62^ ^]^ The network comprised the two distinct main parts: the image process part for the image denoising procedure, and the computation part for the pattern recognition of the processed image. For the image process part, the multi‐layer perceptron model based on the 39×39 input and 17×17 output patches consisting of the synaptic cells was utilized,^[^ ^63^ ^]^ and the computation part involved the four layers connected via the synaptic cell: an input layer consisting of 784 neurons for the denoised images, two processing layers, each with 256 neurons, and an output layer with 10 neurons for the classes of the fashion images. As the synaptic cell for the neural networks, two organic memristors were used, as shown in Figure 5e. To estimate the memory level of the synaptic cell, we trained the synaptic cell using the simple electric stimuli (see Figure S17, Supporting Information) following the gradual set and full reset (GSFR) scheme,^[^ ^64^ ^]^ and tested the memory retention performances of the cell, according to the conductance state (see Figure 5f). The developed synapse clearly showed the forty non‐volatile memory states, and the measured memory states were used in evaluating the cell parameters. In the repeated GSFR cycle tests, our synaptic cell exhibited reliable conductance changes with no errors, indicating that the reliability of our cell is sufficiently superior to avoid programming errors during the training processes (see Figure S17, Supporting Information). Although cell‐to‐cell variations were not considered in estimating the training errors, as in previous studies,^[^ ^56^, ^59^ ^]^ the memristor cell uniformity can be effectively enhanced by engineering the ion injection path.^[^ ^20^, ^42^ ^]^ For training the systems, a software‐based neural network, mirroring the layers of the hardware neural network, was first constructed and optimized for 40 epochs using the 42000 images for denoising and classifying Fashion MNIST images. The optimized weight distributions of the software system were subsequently quantized into the 40‐level conductance states of the artificial synapse, and thereafter, the conductance values were conveyed to the synaptic cell. In testing the system, we employed 1000 images from the Fashion MNIST dataset, and the test images were modified as the noisy patterns by introducing additive white Gaussian noise at a noise level of 50 (see Figure S18, Supporting Information).^[^ ^63^ ^]^ In our optimized system showed the high accuracy (≈92.5%) for recognizing the complex fashion images, and the performance was maintained stably (≈91.1%), irrespective of the additive noise in the images (see Figure 5g). Such performances are close to those of the ideal software with the recognition accuracy of ≈93.0% for the clear images and ≈92.4% for the noisy images (see Figure S19, Supporting Information). However, neural networks built on conventional hardware consisting of only an image computation part exhibited a significant degradation in recognition accuracy when exposed to noisy images (71.1%). This indicates that the process part for denoising the images (see Figure S20, Supporting Information) in the developed system directly contributed to the stable image recognition performance. Considering that the image process part consisting of the synaptic cells with eight conductance states, as estimated in the previous study,^[^ ^11^ ^]^ did not effectively function as a denoising processor, our synapse cell shows high promise for solving complicated and practical problems. Our organic memristor with numerous non‐volatile memory states exhibited the highly superior performances, compared to the previous devices, in the view point of the electrical and mechanical characteristics (see Table S2, Supporting Information),^[^ ^11^, ^54^, ^55^, ^56^, ^57^, ^58^, ^59^, ^65^ ^]^ indicating that our device has a high potential for developing complex wearable neuromorphic systems.

## 3 Conclusion

In conclusion, we demonstrated the flexible memristor with the facilely tunable synaptic conductance close to the biological level to realize the complex wearable neural networks. We explored a physical picture for the filament growth within the polymer media with the different conducting properties, in the organic memristors. The dynamics of the metallic filament growth and the resultant resistive switching characteristics of the device were found to be governed by the polymer conductivity. As the polymer conductivity increased, the filament structure underwent a transformation from the bridge type to the cluster type, resulting in improved linearity in the resistive switching behaviors of the device. We implemented bio‐mimetic synaptic functions, including continuous weights, in the flexible memristors with the low leakge current through the optimization of the polymer conductivity. In our flexibe memristor, the cluster‐structured filaments were gradually grown according to the simple electric stimuli, which leads to the linear resistive switching properties. The device showed the bio‐realistic synaptic plasticity such as STP, LTP, and spike‐depedent plasticity with the filament growth and rupture. In addition, the continuous conductance states with the non‐volatile memory characteristics were effectively achieved in the device attributed to the linearly tunable conductance and the low current level at the HRS. The artificial synapse based on the developed memristor showed the large number of the non‐volatile memory states (forty levels) controlled by the simple electric stimuli. The crossbar arrays of our flexible memristor presented outstanding capabilities for achieving complex hardware neural networks. The fabricated neural network consisting of the developed memristor was reliably operated as the ternary logic operators with high energy efficiency. Furthermore, we demonstrated the smart image recognition system for classifying the complex noisy images stably, utilizing our memristor arrays. Our system stably recognized the fashion images with the high accuracy (≈91.1%), regardless of the noise. This novel concept of achieving bio‐realistic synaptic weights in flexible memristors could serve as an effective strategy for developing the next‐generation computing architecture for wearable smart electronics linked with artificial intelligence.

## 4 Experimental Section

The geometrical morphologies of the devices were analyzed utilizing a surface profiler (DektakXT‐A, Bruker). The electrical property measurements of the devices were performed utilizing a semiconductor parameter analyzer (4200‐SCS, Keithley) connected with an ultrafast *I*–*V* module (4225‐PMU, Keithley). To investigate the electrical properties of the organic memristors, a scanning bias was applied to the top electrode (Ag) while the bottom electrode (Au) was grounded. The filament structures of lateral‐structured memristors were observed utilizing a field‐emission scanning electron microscope (S‐4800, Hitachi).

To fabricate organic memristors with a lateral structure, a glass substrate was first ultrasonically cleansed in acetone, isopropyl alcohol, and deionized water for 20 min. In order to enhance the wettability of the substrate, a 15‐min UVO treatment was conducted at the intensity of 28 mW cm^−2^ in the UVO chamber (AH‐1700, Ahtech LTS Co., Ltd.). A poly (styrenesulfonate)‐doped poly (3,4‐ethylenedioxythiophene) (PEDOT:PSS) (Clevios PVP Al 4083, Heraeus) film was spin‐coated onto the substrate as a polymer medium at the rate of 1500 rpm for 30 s. The polymer film was baked at 110°C for 30 min to remove any residual solvent. The thickness of the PEDOT:PSS layer was ≈180 nm. The PEDOT:PSS film was subjected to UVO treatment at an intensity of 28 mW cm^−2^ for 0 to 100 min in an UVO chamber (AH‐1700, Ahtech LTS Co., Ltd.) to control the polymer conductivity. A 50‐nm gold film was thermally deposited onto the polymer layer at 1 Å s^−1^ under 10^–6^ Torr as an inert electrode. The inert electrode patterns were produced using a hydrophobic polymer (EGC‐1700, 3 M) by a typical transfer‐printing method and a wet‐etching process using a gold etchant (TFA, Transene). On the hydrophobic polymer‐patterned substrate, a 50‐nm thick silver film was prepared for an active electrode using thermal deposition at 1 Å s^−1^ under 10^–6^ Torr. The active electrode was patterned via the lift‐off process to remove the hydrophobic film utilizing a fluorinated solvent (HFE‐7100DL, 3 M). The electrode width and the distance between the electrodes were ≈50 µm and 450 nm, respectively.

A polyethylene naphthalate (PEN) substrate was ultrasonically cleaned in acetone, isopropyl alcohol, and deionized water for 20 min in order to fabricate a flexible organic memristor with a vertical structure. As an inert electrode, a 50 nm gold film was produced on the substrate by thermal evaporation at 1 Å s^−1^ under 10^–6^ Torr. The substrate was subjected to the same UVO treatment as the lateral‐structured memristors to improve the surface wettability. The defined hydrophobic polymer (EGC‐1700, 3 M) patterns on the elastomeric stamp were then transfer‐printed on the substrate to produce selective dewetting regions. For active polymer patterns, the PEDOT:PSS (Clevios PVP Al 4083, Heraeus) solution was spin‐coated at a rate of 1500 rpm for 30 s on the inert electrode with the selective dewetting regions. The polymer patterns were then baked at 110°C for 30 min to remove any residual solvent. The thickness of the polymer pattern was ≈180 nm. The UVO treatment was performed on the PEDOT:PSS film at an intensity of 28 mW cm^−2^ for 50 min in the UVO chamber (AH‐1700, Ahtech LTS Co., Ltd.) in order to modify the polymer conductivity. A 50‐nm silver film was prepared onto the polymer layer using thermal deposition at 1.0 Å s^−1^ under 10^−6^ Torr as an active electrode. The dimension of the active region of the device was measured to be 50 µm × 50 µm.

## Conflict of Interest

The authors declare no conflict of interest.

## Supporting information

Supporting Information

## Data Availability

The data that support the findings of this study are available from the corresponding author upon reasonable request.
