# Supplementary material for: Formation of Cluster‐Structured Metallic Filaments in Organic Memristors for Wearable Neuromorphic Systems with Bio‐Mimetic Synaptic Weight Distributions
Source: Adv Sci (Weinh). 2023 Dec 12;11(9):2307494. doi: 10.1002/advs.202307494 (PMC10916635; doi:10.1002/advs.202307494)
Supplement: Supplementary file 1 — Supporting Information [file ADVS-11-2307494-s001.pdf]

ADVANCED SCIENCE
Open Access

Supporting Information

for *Adv. Sci.*, DOI 10.1002/advs.202307494

Formation of Cluster-Structured Metallic Filaments in Organic Memristors for Wearable Neuromorphic Systems with Bio-Mimetic Synaptic Weight Distributions

*Uihoon Jung, Miseong Kim, Jaewon Jang, Jin-Hyuk Bae, In Man Kang and Sin-Hyung Lee**

Supporting Information

Formation of Cluster-Structured Metallic Filaments in Organic Memristors for Wearable Neuromorphic Systems with Bio-Mimetic Synaptic Weight Distributions

*Uihoon Jung, Miseong Kim, Jaewon Jang, Jin-Hyuk Bae, In Man Kang, and Sin-Hyung Lee**

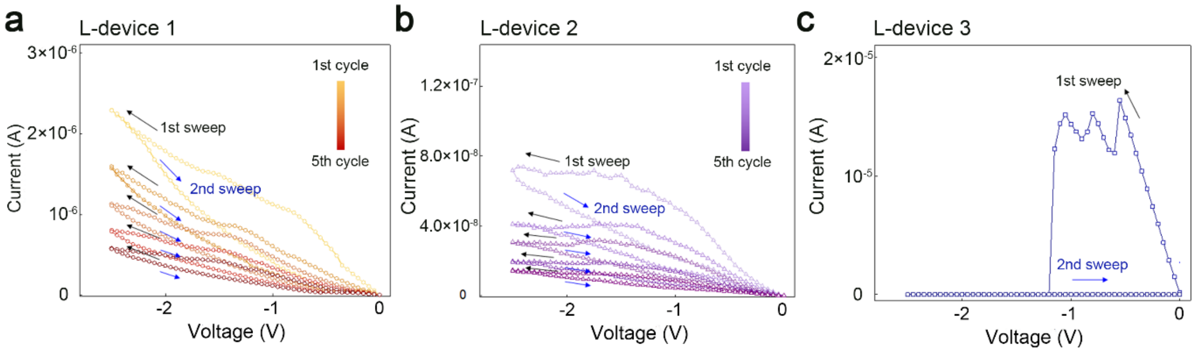

Figure S1. The current−voltage curves for the erasing process of the lateral-structured memristors with the different UVO treatment conditions: (a) L-device 1 with no treatment, (b) L-device 2 with the 50-min treatment, and (c) L-device 3 with the 100-min treatment.

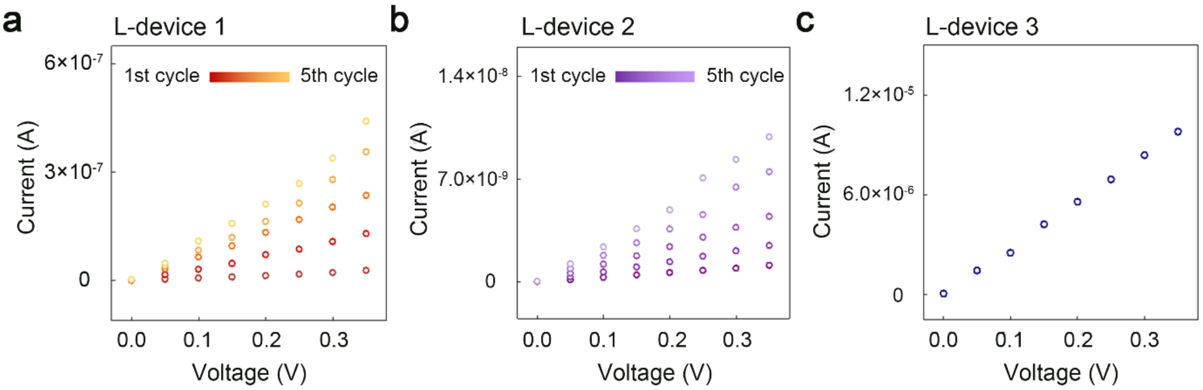

Figure S2. The relationship between the current and voltage values in (a) L-device 1, (b) L-device 2, and (c) L-device 3 at the low resistance states.

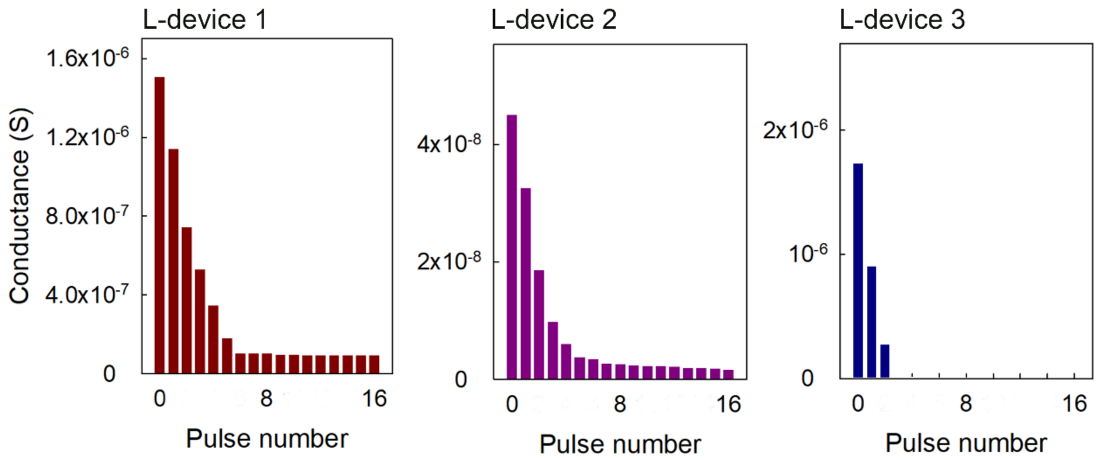

Figure S3. The conductance changes in (a) L-device 1, (b) L-device 2, and (c) L-device 3, during the application of successive -2.2 V-voltage pulses with 100-μs width.

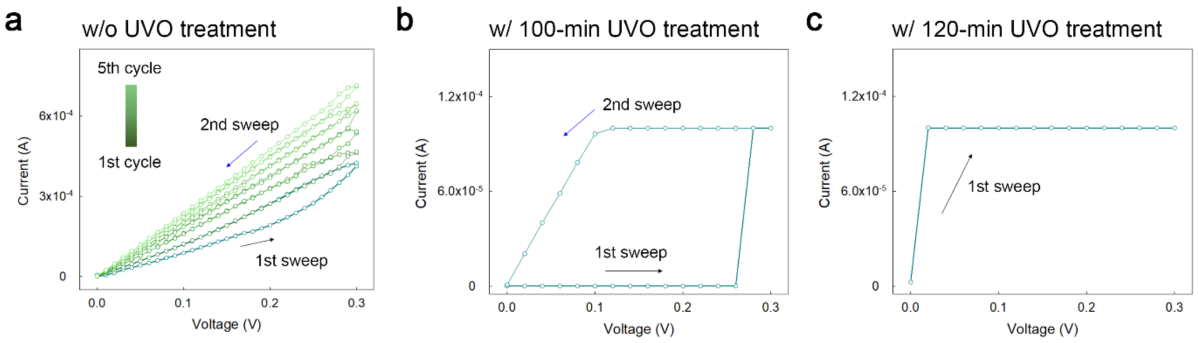

Figure S4. The current−voltage curves for the vertical-structured organic memristors comprising the patterned PEDOT PSS film with the three different UV treatment conditions: (a) with no UVO treatment, (b) with the 100-min UVO treatment, and (c) with the 120-min UVO treatment .

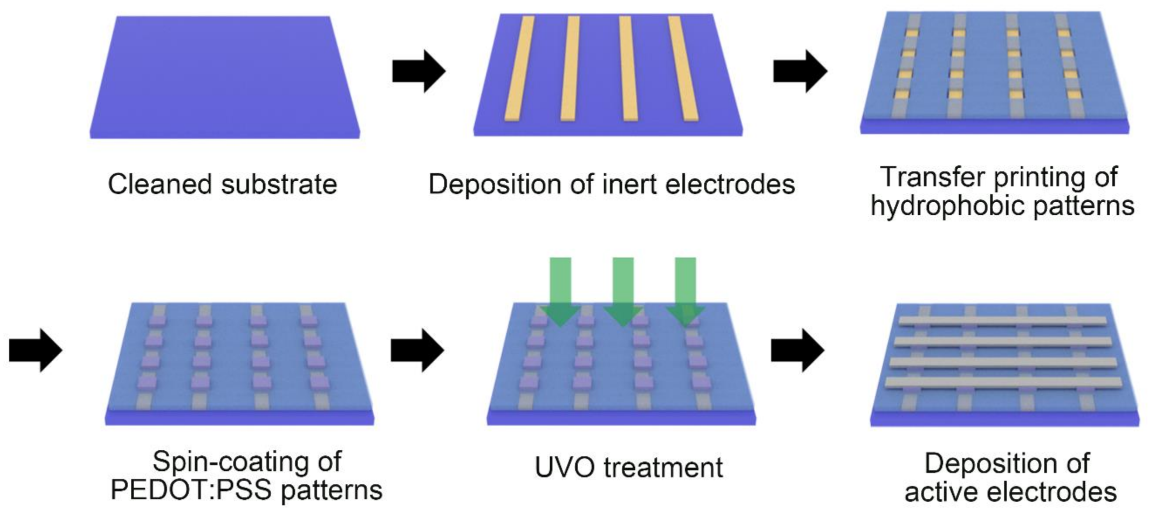

Figure S5. Fabrication steps for the vertical-structured flexible memristor with the patterned polymer (UVO-treated PEDOT:PSS) medium.

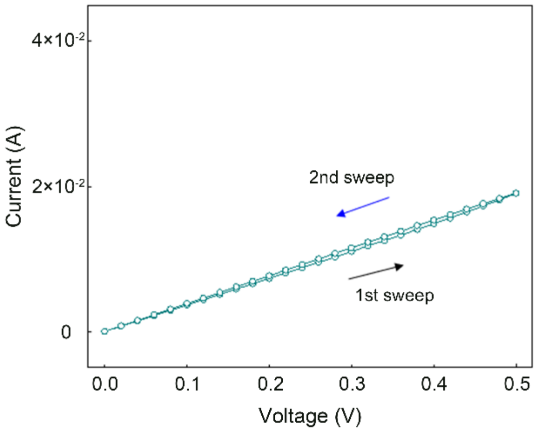

Figure S6. The current−voltage curves for the vertical-structured organic memristor with the non-patterned polymer (UVO-treated PEDOT:PSS) medium.

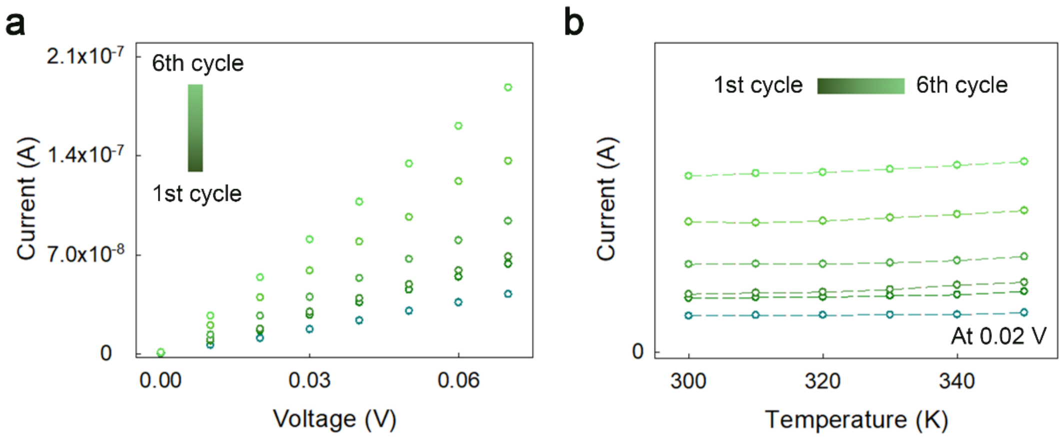

Figure S7. An analysis for confirming a conduction mechanism of the vertical-structured flexible memristors. (a) The relationship between the current and voltage values in the device at the low resistance states (LRSs) (b) Conductance changes of the device at the LRSs, according to temperature from 300 K to 350 K.

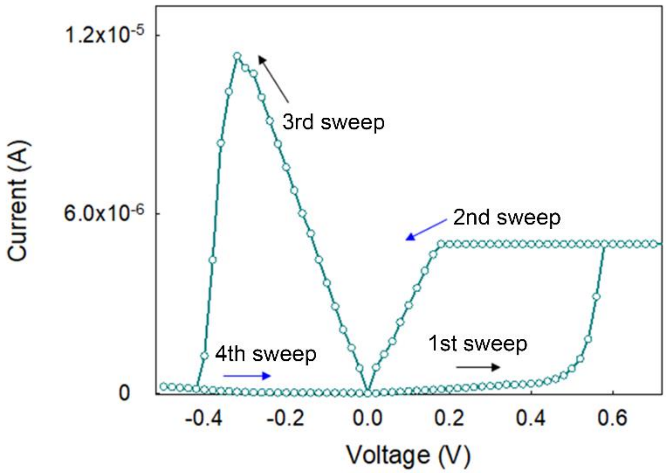

Figure S8. Current–voltage characteristics of the vertical-structured flexible memristor measured under the voltage sweeps ranging from 0 to 0.6 V and from 0 to -0.5 V for writing and erasing processes, respectively.

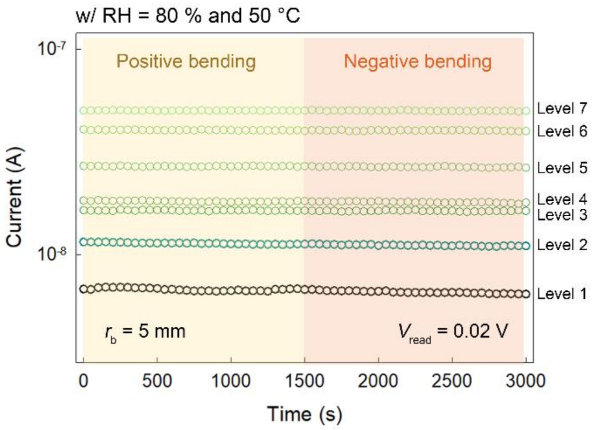

Figure S9. The memory retention performances of the vertical-structure flexible memristor in the harsh environmental condition (with a relative humidity of 80%, at 50 °C). The positive and negative bending stresses with the bending radius of 5 mm was sequentially applied to the device, during the retention test.

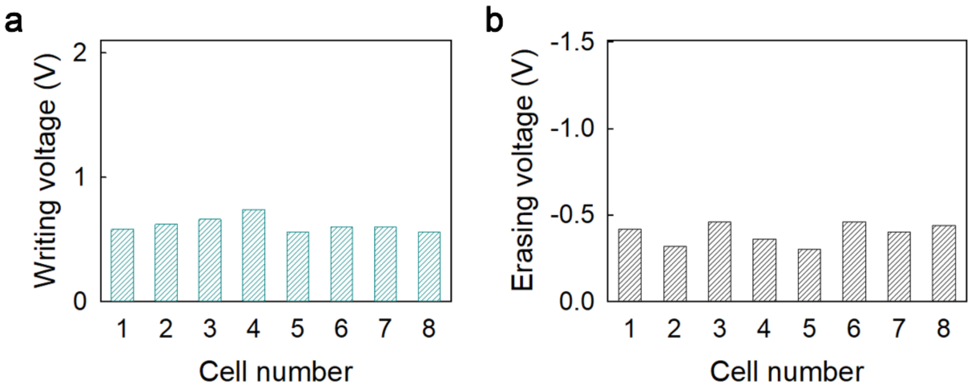

Figure S10. Distributions of the switching (writing and erasing) voltages in the eight different organic memristors prepared on a single substrate.

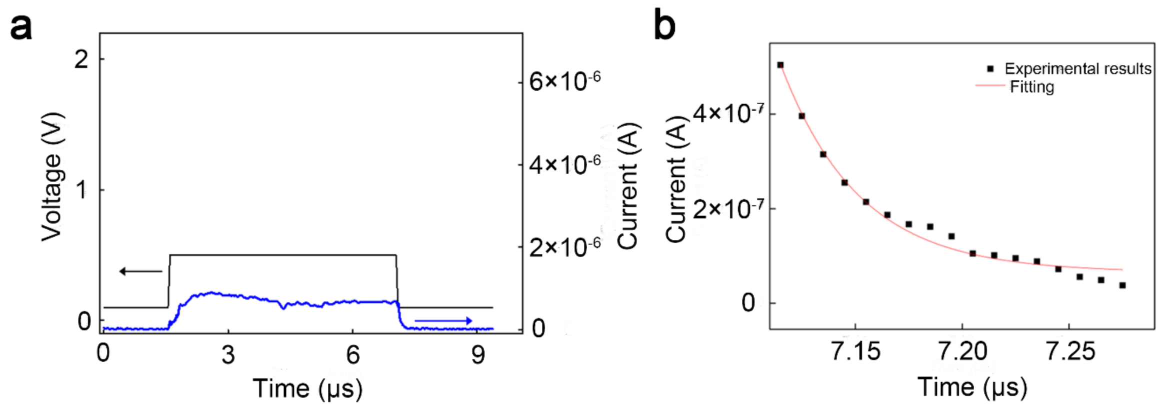

Figure S11. (a) Transient response of the vertical-structured flexible memristor under the 0.5-V voltage pulse. (b) The relaxation characteristics of the device conductance analyzed in Figure S11a. The experimental result was fitted as an exponential decay function.

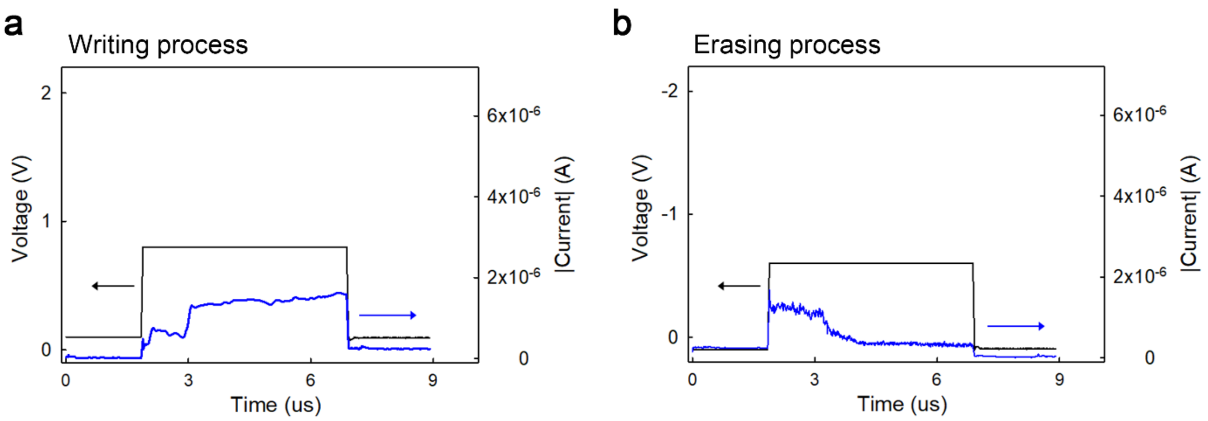

Figure S12. Transient responses of the vertical-structured flexible memristor at the 0.8-V and -0.6-V voltage pulses. (a) The writing and (b) erasing processes of the device under the pulse mode. The writing and erasing times for the device were about 1.1 µs and 1.8 µs, respectively.

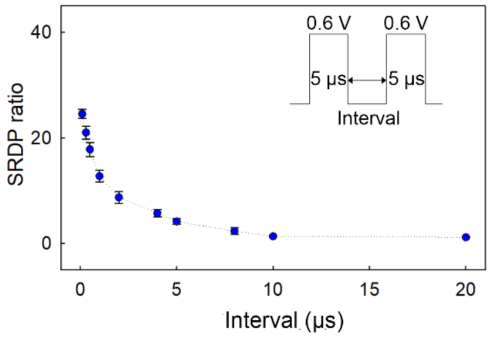

Figure S13. Spike-rate-dependent plasticity of the device, as a function of the time interval between repeated 0.6-V pulses.

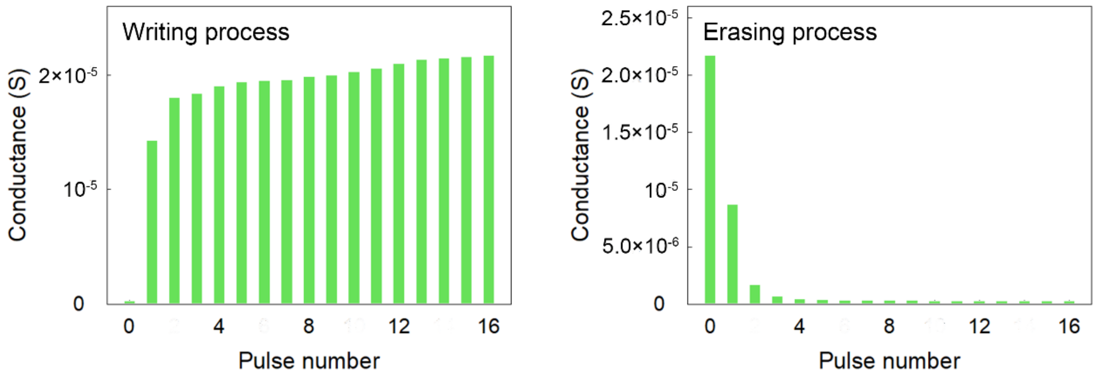

Figure S14. The conductance changes in the vertical-structured flexible memristor, during the application of successive 1.2 V-voltage pulses for writing (-1.0 V-voltage pulses for erasing). The width of each pulse was 1 μs.

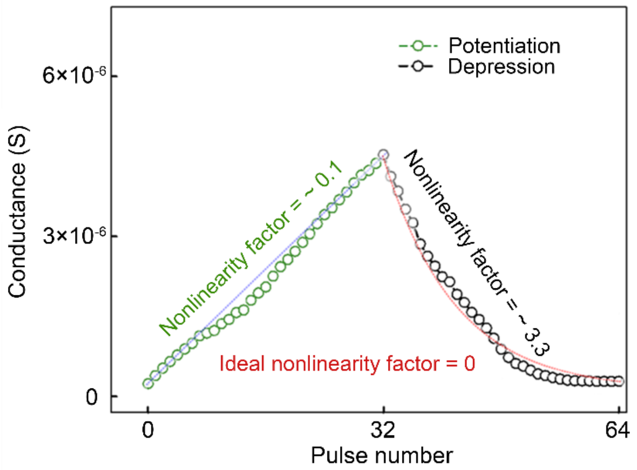

Figure S15. Nonlinearity factors estimated in the vertical-structured flexible memristor. The nonlinearity factor was calculated by the conventional method[1]. The device showed the nonlinearity factor values of ~ 0.1 and ~ 3.3 for the potentiation and depression processes, respectively.

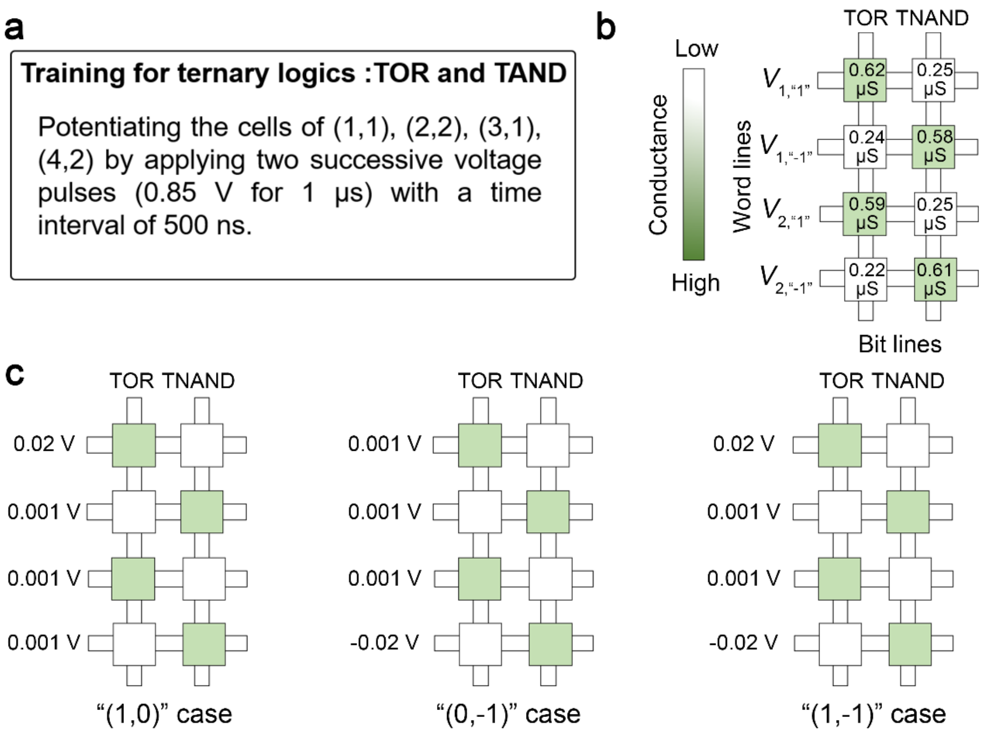

Figure S16. Training of the hardware neural network for the ternary logic (TOR and TAND) operators. (a) A flow chart for training the memristor arrays. In the training process, a floating scheme was utilized. (b) The conductance distributions of the memristor arrays after the training process. (c) The voltage pulses applied to the word lines of the operators, for the representative scenarios of logic inputs.

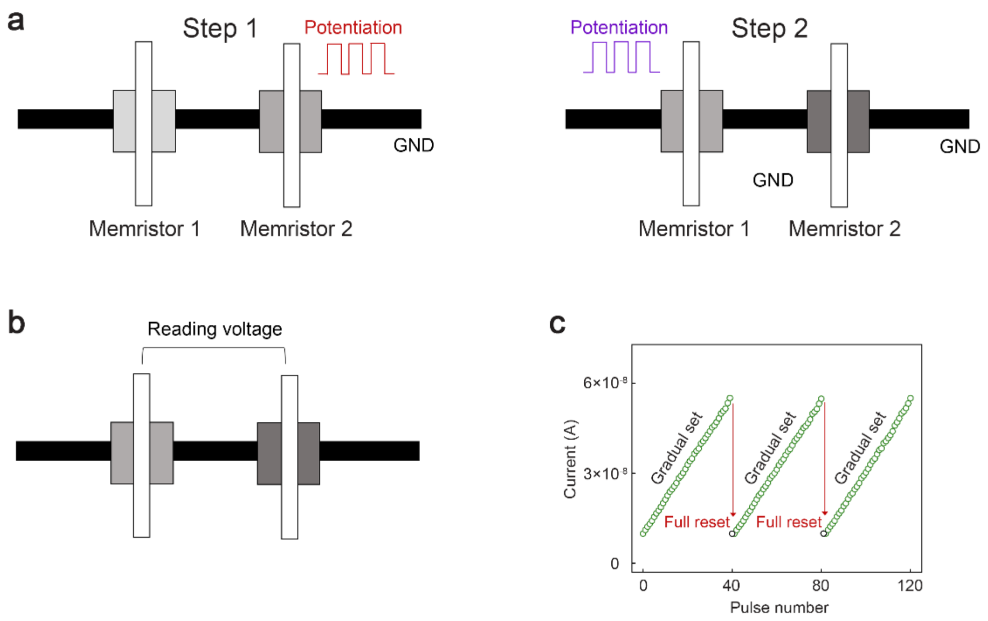

Figure S17. (a) A learning process for achieving the forty conductance states in the artificial synapse involving the two organic memristors (Memristor 1 and Memristor 2). In Step 1, voltage pulses (0.85 V, 1 μs) ranging from 0 to 20 were sequentially applied to Memristor 2. In the step 2, after applying the twenty successive pulses (0.85 V, 1 μs) to Memristor 2, the voltage pulses (0.85 V, 1 μs) with the number from 0 to 19 were biased to Memristor 1. (b) The reading process for checking the synaptic weight in the artificial synapse. (c) The repeated learning processes in the synapse, following the gradual set and full reset scheme.

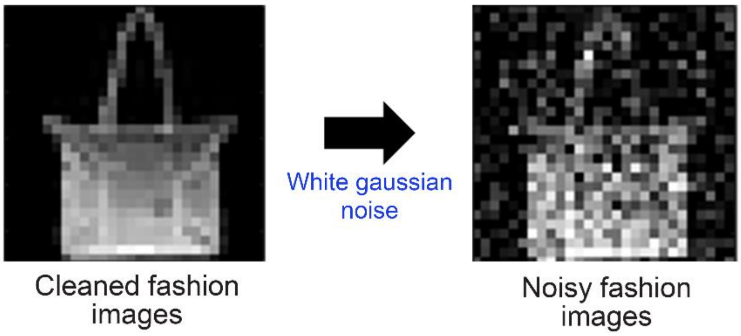

Figure S18. An example image after the process for modifying the Fashion Modified National Institute of Standards and Technology dataset as the noisy patterns. The additive white Gaussian noise was introduced to the images at a noise level of 50.

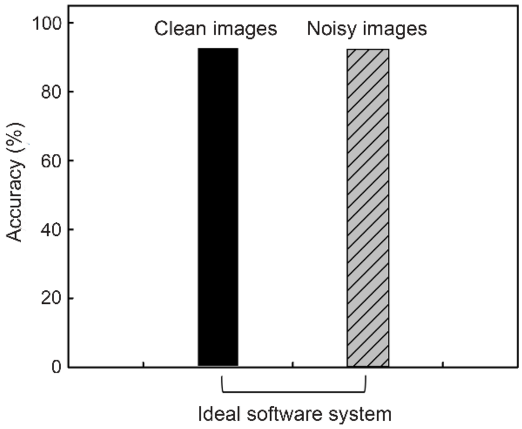

Figure S19. The clean and noisy image recognition accuracy after 40-epoch training in the ideal software system with the same network structure as that in the developed smart hardware neural networks.

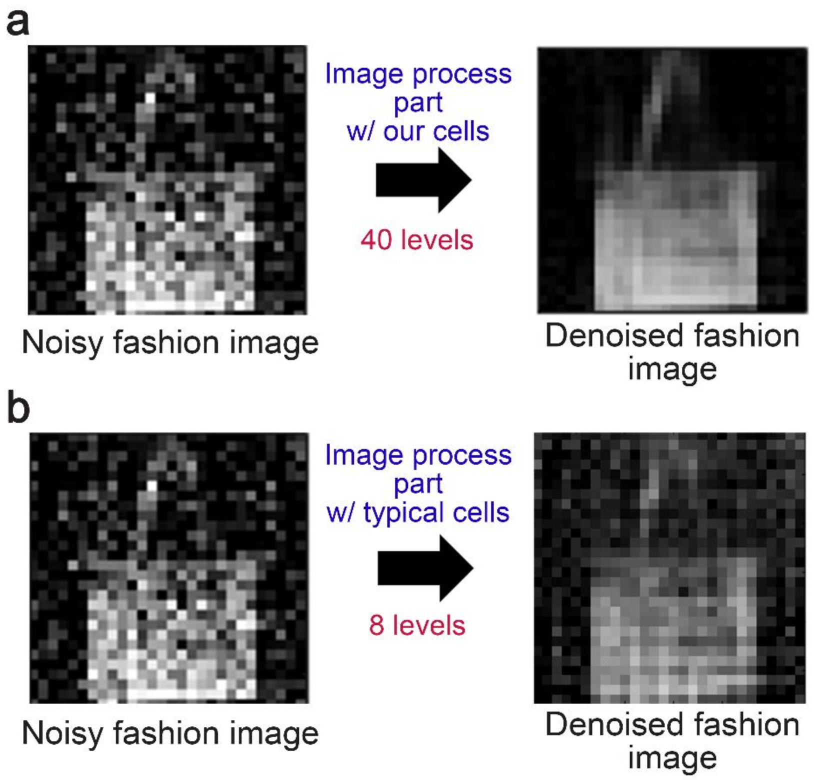

Figure S20. An example images after the denoising process applied to the noisy images within the image process part of (a) the developed neuromorphic system (b) or the system composed of the typical synapse cells with the eight different conductance levels.

Ternary logic input		Ternary logic output	
**Input 1**	**Input 2**	**TOR**	**TAND**
-1	-1	-1	-1
-1	0	0	-1
-1	1	1	-1
0	-1	0	-1
0	0	0	0
0	1	1	0
1	-1	1	-1
1	0	1	0
1	1	1	1

Table S1. A truth table of the ternary logics (TOR and TAND).

Device structure	Write/Erase voltage	On/off ratio	Nonlinearity factor for potentiation	Mechanical flexibility	Non-volatile memory level	Application	Ref.
$Pt/HfAlO_x/TiN$ (single memristor)	1.5 V / -1.0 V	~ 10	~ 10.0	X	2	-	[54]
Ag/Ag doped Np-C_1C/ITO (single memristor)	0.6 V / -0.4 V	~ 3×10	~ 2.7	O	5	Clear image recognition	[55]
Ag/EGC-1700/Au (integrated with external components)	2.0 V / -1.5 V	$\sim 10^1$	~ 0.3	O	8	Clear image recognition	[11]
$Al/TiO_{1.7}/TiO_2/AlO_x$ /Al (single memristor)	1.2 V / -3.0 V	~ 10	~ 0.7	X	6	Clear image recognition	[56]
$Ag/GeO_2/ITO$ (single memristor)	1.2 V / -1.2 V	~10	~ 3.4	O	2	Clear image recognition	[57]
Al/Indigo/Al (single memristor)	4.0 V / -4.0 V	~ 5	~ 5	X	2	-	[58]
Au/Nb (or Zr) dopped TaOx/Pt (single memristor)	5.0 V / -5.0 V	~ 2×10	~ 2	X	2	Clear image recognition	[59]
Ag/CoO/FTO (single memristor)	3.0 V / -3.0 V	~7	~ 0.1	O	2	Clear image recognition	[65]
Ag/UVO-treated PEDOT:PSS/Au (single memristor)	0.6 V / -0.4 V	$\sim 3\times10^2$	~ 0.1 (w/o pulse engineering)	O	40	Ternary logic operator, noisy image recognition	This work

Table S2. Comparison of performances of artificial synapses with a 2-terminal structure.

Supporting Information References

[1] J. Kang, T. Kim, S. Hu, J. Kim, J. Y. Kwak, J. Park, J. K. Park, I. Kim, S. Lee, S. Kim, Y. Jeong, *Nat. Commun.* **2022**, 13, 4040.
